# Supplementary figures and images for: Long noncoding RNA MEG3 suppresses podocyte injury in diabetic nephropathy by inactivating Wnt/β-catenin signaling
Source: PeerJ. 2019 Nov 28;7:e8016. doi: 10.7717/peerj.8016 (PMC6885352; doi:10.7717/peerj.8016)

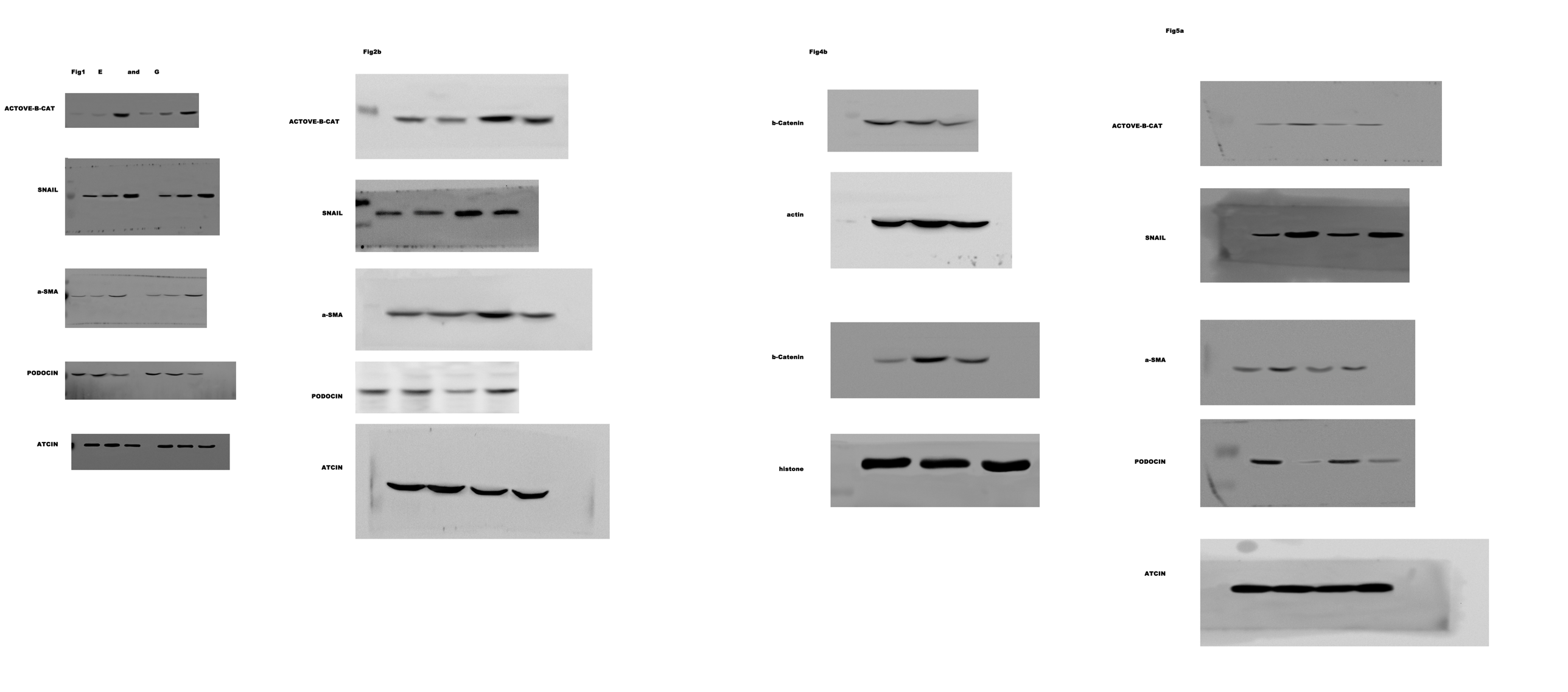

Supplement: Supplemental Information 2 [file peerj-07-8016-s002.jpg]
